# Association of Inflammatory and Oxidative Stress Biomarkers Adjusted by Personal, Psychological, Biochemical, Anthropometric, and Physiological Variables with Global DNA Methylation in a Sample of Mexican Individuals

**DOI:** 10.3390/biom15091271

**Published:** 2025-09-02

**Authors:** Heriberto Jacobo-Cuevas, Jorge Ivan Gamez-Nava, Saúl Ramírez-De los Santos, Carlos Alfonso Mercado-Calderón, Blanca Estela Ríos-González, Juan Manuel Ponce-Guarneros, Aniel Jessica Leticia Brambila-Tapia

**Affiliations:** 1 Group for the Assessment of Prognosis Biomarkers in Autoimmune Disorders, Centro Universitario de Ciencias de la Salud (CUCS), Universidad de Guadalajara, Guadalajara 44340, Mexico; heriberto.jacobo8347@alumnos.udg.mx (H.J.-C.); jorge.gamez@academicos.udg.mx (J.I.G.-N.); 2 Programa de Postdoctorado, Departamento de Psicología Básica, Centro Universitario de Ciencias de la Salud, Universidad de Guadalajara, Guadalajara 44340, Mexico; 3 Instituto de Terapéutica Experimental y Clínica, Departamento de Fisiología, Centro Universitario de Ciencias de la Salud (CUCS), Universidad de Guadalajara, Guadalajara 44340, Mexico; juan.ponce4091@academicos.udg.mx; 4 Departamento de Psicología Básica, Centro Universitario de Ciencias de la Salud (CUCS), Universidad de Guadalajara, Guadalajara 44340, Mexico; saul.rdelossantos@academicos.udg.mx (S.R.-D.l.S.); carlos.mercado5444@alumnos.udg.mx (C.A.M.-C.); 5 Instituto de Investigación en Ciencias Biomédicas (IICB), Centro Universitario de Ciencias de la Salud (CUCS), Universidad de Guadalajara, Guadalajara 44340, Mexico; 6 Unidad de Medicina Familiar No. 92, Instituto Mexicano del Seguro Social, Guadalajara 44340, Mexico; blanca2282@gmail.com

**Keywords:** global DNA methylation, cytokines, inflammation, oxidative stress, sex-specific

## Abstract

Global DNA methylation has been associated with numerous traits and conditions; however, its relationship with inflammation and oxidative stress biomarkers has not been fully elucidated. The objective of this study is to determine the correlation between inflammatory and oxidative stress markers with global DNA methylation after adjusting for personal, psychological, biochemical, anthropometric, and physiological variables in a non-representative sample of the Mexican population. An adult Mexican population was invited to participate and complete a questionnaire with personal and psychological variables. Additionally, anthropometric variables and blood pressure were measured in all the participants. Finally, general blood tests, global DNA methylation analysis, and measurements of inflammatory and oxidative stress markers were performed. A total of 157 participants were included, of which 83 (52.8%) were women, with a median age of 24 years and an age range of 18–58 years. In the comparison between sexes, men showed higher levels of global DNA methylation. In addition, men showed a higher number of correlations with this variable. The bivariate correlations showed low positive correlations of IL-8, IL-10, TNF-α, and 8-isoprostane with global DNA methylation in the total sample. In addition, BMI showed low negative and significant correlations with global DNA methylation in the total, women’s, and men’s samples, while blood pressure showed low negative correlations with global DNA methylation in the men’s sample. Men showed low negative correlations with personal and biochemical variables that were not found in the women’s group. In the multivariate analyses, the psychological variables (SOC-13 comprehensibility, perceived stress, and assertiveness) correlated negatively either in the total, or in men’s or women’s samples, and the daily intake of drugs correlated negatively with methylation in the women’s sample in the bivariate and multivariate analyses. In conclusion, global DNA methylation seems to be related to many variables, including the inflammatory and oxidative stress biomarkers, and this relationship is different in each sex.

## 1. Introduction

Epigenetics refers to the processes that trigger and preserve inheritable gene expression patterns without modifying the DNA sequence itself [1]. DNA methylation is a post-replication modification almost exclusively found in the 5 position of the pyrimidine ring of cytosine in the context of the dinucleotide sequence CpG, producing 5-methylcytosine (5-mC), of which around 29 million are found in the human haploid genome [2,3]. It has been reported that 5-mC represents around 1% of all bases with slight variations in different tissue types, and the majority (60–80%) of CpG dinucleotides throughout mammalian genomes are methylated [4,5]. The sequence symmetry of CpG dinucleotides allows the transmission of DNA methylation marks through cell division, suggesting that DNA methylation marks are part of the cell identity and memory [4]. This CpG can be found grouped in sites known as CpG islands which are clusters of typically non-methylated CpG [6], and these clusters are found in the promoter regions and first exons of around the 65% of all genes, containing about 7% of all CpG sites [4,7,8]. There are around 30,000 CpG islands in the human genome. These regions have a minimum size of 200 base pairs and are mostly non-methylated across all tissues and developmental stages. This corresponds to an open chromatin structure and a potentially active transcriptional state [4].

Alterations in global DNA methylation have been investigated in relation to various diseases and conditions. A large genome-wide DNA methylation study in three large population cohorts identified 5168 age-associated CpG sites that exhibited altered methylation patterns during aging. Among these sites, the majority (61%) showed demethylation in relation to age [9]. This demethylation was characterized by a high concentration of age-methylated CpGs in first exons and upstream transcription start sites and age-demethylated CpGs in other gene regions. These methylation patterns were related to reduced gene activity during aging [9]. Additional studies using peripheral tissue samples, such as blood, have connected numerous conditions to either elevated or reduced global DNA methylation levels. In this sense, one study reported high global DNA methylation levels in a group of anxious individuals when compared with a group of non-anxious individuals [10]. In addition, low global DNA methylation levels have been associated with high body mass index (BMI) in system lupus erythematosus (SLE) patients, which was also related to the downregulation of the enzyme DNA methyltransferase 1 (DNMT1) [11]. DNA hypomethylation has also been associated with a higher likelihood of developing breast cancer and the presence of the disease [12,13]. Moreover, there is a study reporting a negative correlation between placental global DNA methylation and maternal mean blood pressure in mothers without a family history of hypertension [14].

Regarding studies searching for gene-specific methylation, a meta-analysis conducted on essential hypertension reported different methylation patterns of the studied genes. Some genes showed hypermethylation, whereas others showed hypomethylation, suggesting an aberrant epigenomic modulation of candidate genes involved in hemodynamics and homeostasis [15]. Similarly, an recent review on chronic lymphocytic leukemia (CLL) found that gene-specific hypermethylation and hypomethylation were related to prognosis and survival [16]. Collectively, these findings suggest that both global DNA and gene-specific methylation play a role in the development and prognosis of chronic diseases. On the other hand, global DNA methylation has been investigated in relation to pro-inflammatory and anti-inflammatory cytokines in the context of specific diseases and conditions [17,18]. Previous studies have shown that inflammation alters DNA methylation patterns in adults with sepsis [18]. Additionally, differentially methylated sites have been identified in newborns from mothers with severe-SARS-CoV-2 infection [17]. Moreover, DNA hypomethylation has been associated with poorly controlled type 2 diabetes, and global DNA methylation was found to be negatively correlated with inflammatory and oxidative stress markers in this population [19].

However, the search for a correlation of these cytokines, along with oxidative stress markers, with global DNA methylation in a sample of the Mexican adult population, adjusted by personal, psychological, and anthropometric variables, has not been performed. Therefore, the aim of this study is to examine the associations of a comprehensive set of personal, psychological, biochemical, anthropometric, and physiological variables, along with inflammatory and oxidative stress biomarkers, with global DNA methylation and to evaluate these associations through multivariate analyses in the total sample and stratified by sex.

## 2. Subjects and Methods

### 2.1. Ethical Considerations and Study Population

The study was conducted in accordance with the guidelines of the Declaration of Helsinki and was approved by the Research and Ethics committee of the Health Sciences University Center (approval number CI-06123, approved on 25 September 2024). All participants provided informed consent.

### 2.2. Subjects

The selection criteria were as follows: (a) age from 18 to 60 years, (b) not pregnant, (c) not genetically related to another participant of the study (i.e., siblings, cousins). The exclusion criterion was the absence of measurement of any variable.

### 2.3. Study Design: This Is an Observational, Cross-Sectional Study

The variables of interest were measured at a single time point without any intervention or manipulation by the researchers. This design allows for the identification of associations between variables but does not establish causal relationships, as temporal sequencing cannot be determined.

### 2.4. Procedures

The invitation was carried out through announcements shared via social media platforms (such as WhatsApp Platforms, Inc., Menlo Park, CA, USA) and printed brochures. University students and staff from the Health Sciences University Center were personally invited. The advertisement was circulated over a two-month period, coinciding with the study duration. The research team ensured that all participants met the inclusion criteria. Those who agreed to participate were scheduled to meet in a computer lab at the University of Guadalajara, where they signed an informed consent form and completed an electronic questionnaire (using Google Forms) that gathered personal information. Prior to completing the questionnaire, anthropometric measurements—including body mass index (BMI) and waist-to-hip ratio (WHR)—were taken. Additionally, systolic (SBP) and diastolic (DBP) blood pressure were measured on the left arm using an Omron upper-arm blood pressure monitor (model HEM-7320, Omron corporation, Kyoto, Japan). To ensure accuracy, the readings from this device were cross-validated against those obtained using a standard manual blood pressure cuff in both the first and last participants of the study.

#### 2.4.1. Personal and Psychological Variables

##### Personal Variables

The personal and sociodemographic variables assessed included: sex, age, education level, employment status, having a romantic partner, parental status, socioeconomic level, daily time spent on physical activity, daily hours of free time, and frequency of illegal drug, alcohol, and tobacco use. The latter was evaluated using a five-point scale ranging from “never” to “four or more times per week”. The presence of 27 illnesses within the past six months was assessed through self-report: diabetes mellitus (type 1 or type 2), thyroid problems, allergies (asthma, conjunctivitis, etc.), high cholesterol, gastritis, colitis or irritable bowel syndrome (IBS), migraine/tension headache, skin problems (acne, neurodermatitis), gastrointestinal infections, ulcer disease (gastric or intestinal ulcer), sinusitis, kidney disease (renal failure, kidney stones), anorexia/bulimia, depression requiring medication, anxiety requiring medication, heart attack/angina, rheumatic diseases (rheumatoid arthritis, lupus, ankylosing spondylitis), heart failure, stroke or cerebral infarction, chronic infections (HIV, tuberculosis, long COVID, etc.), cancer (breast, cervical, prostate, skin), leukemia or lymphoma, advanced cancer (metastasis), varicose veins (venous insufficiency, varicose veins), liver disease (hepatitis, cirrhosis, fatty liver), chronic lung disease, respiratory infections (including COVID), hemiplegia (paralysis), and any other chronic illness (requiring medication or continuous management). We additionally measured the number of different drugs taken daily as the variable “daily drug intake”.

##### Psychological Variables

To measure physical symptoms we used the Somatic Symptoms Scale-8 (SSS-8) [20], emotional intelligence abilities including assertiveness, emotion identification, and self-motivation were measured with 5 to 6 items for each subscale of the Trait Emotional Intelligence Questionnaire (TEIQue) (Supplementary File S1) [21], the sense of coherence was measured with the Sense of Coherence Scale (SOC-13) through 13 items that cover three core dimensions: comprehensibility, manageability, and meaningfulness [22], positive emotions were evaluated with the Positivity Self-Test (PST) [23], to measure anxiety symptoms we used the Generalized Anxiety Disease Scale (GAD-7) [24], adverse life events were measured with the Cumulative Lifetime Adversity Measure Scale which measured 37 different lifetime adverse events (traumatic events) with 4 different frequency options (from never to more than 2 times) [25], to measure perceived stress we used the Perceived Stress Scale (PSS-10) [26,27], depressive symptoms were evaluated with the Patient Health Questionnaire (PHQ-9) [28], and psychological wellbeing was evaluated with Ryff’s Psychological Well-Being Scale (PWBS), which is based on six theoretical dimensions of psychological well-being: self-acceptance, positive relations with others, autonomy, environmental mastery, purpose in life, and personal growth [29,30].

##### Lifestyle Scales

Sleep satisfaction was assessed using the first item of the OVIEDO sleep questionnaire, with response options ranging from 1 (very dissatisfied) to 7 (very satisfied). Sleep quality was evaluated through the second component of the questionnaire, which comprises five items, each rated on a scale from 0 (poor quality) to 4 (excellent quality) [31], and to evaluate the quality of food intake we used a mini-survey (Mini-ECCA survey) to evaluate this variable in the Mexican population [32].

#### 2.4.2. Biological Sample Collection

Fasting blood samples were obtained by venipuncture in all participants during the early morning hours (the participants had fasted for at least 8 h) by personnel or the research team. After the samples were obtained, the biochemical analyses were performed.

#### 2.4.3. Biochemical Variable Measurement

Analysis of biochemical variables was performed on blood samples obtained from all participants to quantify the following biochemical parameters: (1) complete blood count test (including hemoglobin, hematocrit, platelets, leukocytes, and their subpopulations); (2) total cholesterol and triglycerides; and (3) blood chemistry (including glucose and urea). The measurements were performed with the electronic impedance variation method (HORIBA ABX Micros ES 60, Hematology Analyzer) for the blood count tests and colorimetry (H-100 Automated Clinical Chemistry Analyzer) for all biochemical tests.

#### 2.4.4. Serum Levels of Inflammatory and Oxidative Stress Biomarker Analysis

The quantitation of serum biomarkers levels was performed using the ELISA technique, following the manufacturer’s instructions for each assay. Serum samples were cryopreserved at −80 degrees Celsius until their processing. To quantify TNF-α, IL-8, IL-6, IL-1β, IL-10, 8-isoprostane, and 8-hydroxy-2′-deoxyguanosine, we used catalog numbers MBS175820, MBS763092, MBS021993, MBS263843, MBS764410, MBS3802509, and MBS267161, respectively. All kits were obtained from Mybiosource (Mybiosource, Inc., San Diego, CA, USA).

#### 2.4.5. DNA Extraction

Genomic DNA was extracted from the leukocytes in peripheral blood samples using the modified Miller and CTAB/DTAB method. DNA concentration (absorbance at 260 nm) and purity (ratio of absorbance at 260 nm to absorbance at 280 nm) were measured using a NanoDrop spectrophotometer (NanoDrop Technologies Inc., Thermo Fisher Scientific, Wilmington, DE, USA). DNA was diluted in Tris-EDTA buffer to a 100 ng/μL concentration.

#### 2.4.6. Inmmunoquantification of Global DNA Methylation

Global DNA methylation was assessed in 100 ng of leukocyte-derived DNA, in duplicate. Methylated cytosine (5-mC) was identified using specific capture and detection antibodies and subsequently quantified via a standard curve and a colorimetric method by measuring absorbance at 450 nm with a microplate spectrophotometer, Multisca^TM^ FC (Thermo Fisher Scientifi^TM^, Life Technologies, Waltham, MA, USA). The level of DNA methylation is directly proportional to the optical density (OD) recorded. Global DNA methylation quantification was performed as per the manufacturer’s instructions using the Methylflash Methylated DNA Quantification Kit, catalog number P-1030 (Epigentek, Farmingdale, New York, NY, USA).

#### 2.4.7. Statistical Analysis

To describe the continuous variables, we used mean and standard deviations when the distribution was parametric and median and ranges when it was non-parametric. To describe categorical variables, we used frequencies and percentages. To compare socio-demographic variables between sexes, we used a chi-squared test for qualitative variables and Student’s t-test or the Mann–Whitney U test for quantitative ones (depending on whether the distribution was parametric or non-parametric, respectively). To determine the correlation of the global DNA methylation values with each personal, psychological, biochemical, anthropometric, and physiological variable, along with inflammatory and oxidative stress biomarkers, we used the Pearson and Spearman correlation tests, depending on whether the distribution of the data was parametric or non-parametric, respectively. To identify the independent variables most strongly associated with the percentage of global DNA methylation, a multiple linear regression analysis was performed using the stepwise method. The dependent variable (global DNA methylation) was continuous and not normally distributed, while the independent variables included continuous, dichotomous, and ordinal types. The stepwise approach was chosen to build a model in which all retained predictors were statistically significant. Analyses were conducted on the full sample and stratified by sex. This method allowed for control of potential confounding effects and provided adjusted significance values for each variable included in the model. All analyses were performed with the software SPSS v.25, and a *p*-value < 0.05 was considered significant.

## 3. Results

A total of 176 persons agreed to participate and were included in the study. Nineteen participants were excluded because they did not complete the measurements, therefore a total of one hundred and fifty-seven participants were finally included in the analyses. Detailed descriptive data of participants and comparison between the sexes are summarized in Table 1. Among the included participants, 83 (52.8%) were women and the median age was 24 years with a range from 18 to 58 years. Approximately 67% of participants were employed, and 70.5% reported a monthly income within the lower-middle to upper-middle range (MXN 7474 to MXN 17,308). We observed that women had a lower monthly income and lower education level than men. Regarding health and psychological variables, women reported higher levels than men in total number of illnesses. On the other hand, we observe that men had a higher frequency of use of alcohol and a higher use of illicit substances. Women exhibit higher levels of somatic symptoms (1.75 vs. 1.50, *p* = 0.004) and stress (1.62 vs. 1.39, *p* = 0.048) than men, whereas the latter show higher levels of positive emotions (2.92 vs. 2.64, *p* = 0.015), comprehensibility (4.80 vs. 4.20, *p* = 0.033), and manageability (4.49 vs. 4.09, *p* = 0.078).

With respect to blood analyses, which included complete blood count and clinical chemistry, we observed that women had a significantly higher platelet count (236.5 vs. 209.4 (1 × 103/uL), *p* < 0.001), whereas men exhibited higher levels of hemoglobin, glucose, and global DNA methylation percentage (14.5 vs. 12.7 (g/dL), *p* < 0.001; 92.3 vs. 83.8 (mg/dL), *p* < 0.001; and 0.50 vs. 0.43%, *p* = 0.045, respectively). Regarding the quantification of serum levels of inflammatory and oxidative stress biomarkers, no significant sex differences were observed, except for interleukin-6 (IL-6), which was found to be higher in women compared to men (6.2 vs. 4.2 pg/mL, *p* = 0.006). With respect to anthropometric measures, BMI did not differ between sexes, whereas both systolic and diastolic blood pressure were significantly higher in men (*p* < 0.001 and *p* = 0.025, respectively). Additionally, WHR was higher in men, with a median of 0.87 compared to 0.78 in women (*p* < 0.001).

Cronbach’s alpha coefficients for all scales and subscales exceeded 0.6, suggesting that the measures were sufficiently reliable for analytical purposes.

In Table 2, we present the results of the total and sex-stratified bivariate correlation analyses. This table presents the variables that showed statistically significant associations, observed in at least one study group, with the percentage of global DNA methylation. In the total sample we found a very low negative correlation—meaning that, as global DNA methylation increases, the negative correlated variables decrease—between global DNA methylation and age, having children, daily drug intake, granulocytes, diastolic BP, and BMI. Among women, a significant negative low correlation was observed only with daily drug intake and BMI. Whereas in men, significant negative correlations were found with the following variables: age, having a romantic partner, having children, employment, hemoglobin, granulocytes, triglycerides, systolic and diastolic BP, BMI, and WHR. Additionally, in the total sample low significant positive correlations were found with monthly income, the amount of free time, 8-isoprostane, TNF-α, IL-8, and IL-10, and in men, significant low positive correlations were also observed with the amount of free time and serum levels of TNF-α, IL-8, and IL-10.

In the overall multivariate analysis, global DNA methylation was positively associated with interleukin-8 (IL-8), free time, and male sex, while BMI, diastolic blood pressure, and assertiveness were negatively associated. In the female subsample, positive associations were observed with 8-isoprostane and SSS-8 scores, whereas BMI, daily drug intake, PSS-10 scores, SOC-13 comprehensibility, and smoking frequency showed negative associations. In the male subsample, IL-8, monthly income, and free time were positively associated with global DNA methylation, while SOC-13 comprehensibility, diastolic blood pressure, and having a romantic partner were negatively associated (Table 3).

The multivariate regression models demonstrated good explanatory power, with the following fit statistics: for the total sample, R = 0.555 and R^2^ = 0.308, indicating that 30.8% of the variance in global DNA methylation was explained by the predictors; for women, R = 0.587 and R^2^ = 0.344, explaining 34.4% of the variance; and for men, R = 0.776 and R^2^ = 0.602, explaining 60.2% of the variance.

## 4. Discussion

In the comparison of the studied variables between sexes, we observed that most variables were similar between them, with the exception of some variables that have shown significant differences between sexes in previous reports, including a higher number of illnesses and somatization in women when compared with men [33,34,35], as well as higher levels of negative psychological variables (including depressive and perceived stress symptoms) and lower levels of positive psychological variables (including positive emotions and autonomy) in women than in men [33,36,37,38]. Likewise, the higher values of WHR, blood pressure, and cholesterol in men have also been found by our research group and other research groups in the Mexican population [39,40].

With reference to the inflammatory markers, we observed that IL-6 showed higher levels in women than in men, and this finding is related to the higher levels of the inflammatory marker C-reactive protein found in women compared to men in a sample of a healthy Mexican population [41]. In addition, both biomarkers have shown a positive correlation between them [42]. Regarding blood analyses we found that women had a significantly higher platelet count than men, a finding previously reported by Butkiewicz et al. [43], and men showed higher hemoglobin levels, which is consistent with prior evidence [44].

With respect to global DNA methylation levels, we observed that men exhibited higher levels of global DNA methylation, and these findings coincide with a previous report in which higher methylation levels were found in men [45]. This is also shown in another report in which different methylation patterns were found between the sexes [46]. These differences have been attributed to chromosome differences [45], and it has been proposed that the sex differences in traits and conditions could be related to the differences reported in the methylation patterns between the sexes [46].

Regarding the bivariate correlations in the total sample and segmented by sex, we observed that men showed a higher number of significative correlations with global DNA methylation, among which some inflammatory and oxidative stress markers showed positive correlations with global DNA methylation, including TNF-α, IL-8, and IL-10. All of them also showed significative low positive correlations in the total sample in which the oxidative stress marker 8-isoprostane also showed a low positive correlation. When adjusting by the other variables in the multivariate analysis, we observed that IL-8 remained positively correlated in the total and men’s sample, while the oxidative stress biomarker 8-isoprostane showed a significative positive correlation in the multivariate analyses in the women’s sample. These findings coincide with both hypotheses, suggesting a link between inflammation and oxidative stress and global DNA methylation in both sexes but with slight differences. In line with these findings, the study of Lorente-Sorolla et al. [18] showed that, in the context of sepsis, the increase in the cytokines IL-8 and IL-10 coincided with a change in methylation patterns with a higher increase in the hypermethylated sites than the hypomethylated sites. Our results also coincide with a previous report showing an association between oxidative stress markers and DNA methylation and the incidence and development of type 2 diabetes [47]. However, a recent report on poorly controlled type 2 diabetes mellitus patients showed that hypomethylation was associated with the disease, disease duration, and diabetic foot ulcers. In addition, inflammatory and oxidative stress biomarkers were negatively correlated with global DNA methylation [19]. These results are opposite to the correlations found in the present study and could be attributed to the population and the biomarkers included in that study that differ from the population and biomarkers of our study. However, the findings of the present study suggest that inflammatory and oxidative stress processes are positively correlated in both sexes with global DNA methylation in a non-representative sample of Mexican adults and showed a higher correlation in men. Therefore, inflammatory and oxidative stress biomarkers could be used as potential covariates in case–control and correlation studies between global DNA methylation and specific conditions or molecules and sex-segmented analyses should also be performed.

Other variables that showed significant correlations with global DNA methylation included: age, blood pressure, BMI, and daily drug intake. Most of these variables showed low negative and significative correlations with global DNA methylation, mainly in the total and men’s samples, while BMI and daily drug intake were the only variables significantly correlated in women in the bivariate and multivariate analysis.

In this regard, it has been reported that age is associated with lower DNA methylation levels [9], a finding that may be related to reduced gene activity during aging, however, this negative correlation did not appear in the multivariate analyses, which suggests that this association could be mediated by other covariates such as BMI. In this sense the negative correlations between BMI and global DNA methylation coincide with a previous report demonstrating an inverse relationship between DNA methylation and BMI in SLE patients and SLE mouse models [11], which was related to a downregulation of the DNMT1 enzyme. In addition, these results are consistent with a previous longitudinal epigenome-wide association study (EWAS), which demonstrated that specific methylated CpG sites were associated with obesity as a consequence of the condition [48]. These findings, along with those presented here, suggest that obesity plays a role in DNA methylation, which could also be related to obesity-associated diseases.

In relation to blood pressure, we observed that SBP and DBP were negatively correlated with global DNA methylation in the total and men’s samples, and only DBP remained significantly correlated with global DNA methylation in the multivariate analyses of the total and men’s samples. These results coincide with a report showing a negative correlation between placental DNA methylation and maternal mean blood pressure in mothers without a family history of hypertension [14]. Although no other related reports on the relationship between DNA methylation and blood pressure were found, these coincidences suggest that there could be a causal relationship between blood pressure and global DNA methylation, although the direction of this relationship remains to be elucidated.

The negative correlation between daily drug intake observed in the bivariate analyses (in both the total sample and the women’s subsample), as well as in the multivariate analysis for the women’s group, is consistent with a previous report [49]. That study found a higher number of differentially methylated positions in patients with multiple sclerosis compared to controls. These epigenetic changes were more evident in the untreated group, which showed a greater proportion of both hyper- and hypomethylated positions [49]. These findings suggest that drug intake could modify DNA methylation, probably by diminishing inflammation, and these modifications could be related to less global DNA methylation in blood cells. However, analyses of specific drugs, sites in genes, as well as specific genes should be performed.

With respect to the correlations found between global DNA methylation and psychological and behavioral variables, we observed that free time showed low positive but significant correlations with global DNA methylation in the total and men’s samples in the bivariate and multivariate analyses. These results have not been previously reported and suggest that behavioral variables could modify global DNA methylation and gene expression and deserve further investigations. These correlations are in line with the observed correlations of positive and negative psychological variables in the multivariate analyses of the total, men’s, and women’s samples. Interestingly, these correlations were not observed in the bivariate analyses but emerged after adjusting for covariates in the multivariate models. Among the associated variables, the subscale of comprehensibility of the SOC-13 scale was negatively correlated with global DNA methylation in men’s and women’s multivariate analyses. In addition, the subscale of assertiveness of the TEIQue scale was negatively correlated with global DNA methylation in the multivariate analysis of the total sample. Finally, perceived stress was also negatively correlated with global DNA methylation in the multivariate analysis of the women’s sample. Although no previous studies investigating these specific correlations were found, the present results are consistent with reports linking DNA methylation to psychological variables. For instance, one study reported increased DNA methylation in individuals with post-traumatic stress disorder compared to controls [50], while another found epigenetic and behavioral alterations in male and female offspring of rats exposed to paternal stress prior to conception [51]. These results suggest a relationship between stress and psychological variables and DNA epigenetic modifications; however, the causal relationship should be further investigated in longitudinal studies that include gene-specific and region-specific analyses.

Other variables associated with global DNA methylation, including somatization, having a romantic partner, and monthly income in the multivariate analyses of men’s and women’s samples, have not been previously researched and should be further investigated. However, the negative borderline correlation between global DNA methylation and smoking frequency in the multivariate analyses of the women’s sample is in line with a previous report showing lower DNA methylation levels in LINE-1 repeat elements in smokers and vapers [52].

The main limitations of the study are the small sample size, which increases the random bias and diminishes the precision in the correlations found, particularly when multiple comparisons and multivariate analyses with many variables are performed. Therefore, the results should be considered with caution and can be an antecedent for further larger studies. In addition, the transversal nature of the study impedes us from determining potential causal relationships. However, the inclusion of a large number of variables diminishes the confusion bias by adjusting the confounding factors or covariables. In addition, the sex-segmented analyses permitted more specific associations related to sex.

In conclusion, global DNA methylation was positively correlated with inflammatory and oxidative stress biomarkers and negatively correlated with a wide range of personal, psychological, anthropometric, and physiological variables. Many of these associations remained significant in the multivariate analysis. Among them, male sex, BMI, SBP, and DBP appeared to be the most relevant variables to control in association studies, as they are easy to measure and do not require extensive resources.

Additionally, variables such as daily free time, perceived stress, and the SOC-13 subscale of comprehensibility may also influence these associations in analytical studies. Furthermore, the cytokines TNF-α, IL-8, and IL-10 and the oxidative stress marker 8-isoprostane showed weak but significant positive correlations with global DNA methylation and could represent important confounding factors to consider.

Finally, our findings suggest that global DNA methylation is associated with inflammatory and oxidative stress processes, particularly in men. However, the direction of this causal relationship, as well as its connection to specific DNA regions and genes, should be further explored in larger, longitudinal studies incorporating both epigenome-wide association studies (EWASs) and gene-specific analyses.

## Figures and Tables

**Table 1:** Comparison of studied variables between sexes.

Variables	Total	Female	Male	*p*
	N = 157	N = 83	N = 74	
**Sociodemographics**				
Age (years), median (range)	24.0 (18–58)	23.0 (18–58)	25.5 (18–54)	0.535
Highest education level, n (%) - Middle school - High school - Bachelor’s degree - Master’s degree - Doctorate (Ph.D.)				
	7 (4.5)	4 (4.8)	3 (4.1)	
	66 (42.0)	41 (49.4)	25 (33.8)	
	66 (42.0)	35 (42.2)	31 (41.8)	0.019
	11 (7.0)	2 (2.4)	9 (12.2)	
	7 (4.5)	1 (1.2)	6 (8.1)	
With a romantic partner, n (%)	88 (56.1)	42 (50.6)	46 (62.2)	0.145
Having children, n (%)	44 (28.0)	22 (26.5)	22 (29.7)	0.653
Employed, n (%)	105 (66.9)	52 (62.7)	53 (71.6)	0.233
Monthly income (MXN), n (%) - Low - Lower-middle - Upper-middle - High - Very high				
	8 (5.1)	7 (8.4)	1 (1.4)	
	48 (30.6)	33 (39.8)	15 (20.3)	
	61 (38.8)	33 (39.8)	28 (37.7)	
	25 (15.9)	6 (7.2)	19 (25.7)	<0.001
	15 (9.6)	4 (4.8)	11 (14.9)	
Free time (hours), median (range)	4.0 (0.0–12.0)	4.0 (0–12.0)	4.0 (0–12.0)	0.077
Daily exercise (hours), n (%) - 0 to 0.5 - 1.0 to 1.5 - 2.0 to 2.5 - >3				
	64 (40.7)	35 (42.2)	29 (39.2)	
	59 (37.6)	34 (41.0)	25 (33.8)	
	24 (15.3)	10 (12.0)	14 (18.9)	0.476
	10 (6.4)	4 (4.8)	6 (8.1)	
Weekly dietary supplement use, n (%) - Never - Less than once - 1 to 2 - 3 to 4 - Daily				
	87 (55.5)	41 (49.4)	46 (62.1)	
	31 (19.7)	16 (19.3)	15 (20.3)	
	12 (7.6)	8 (9.6)	4 (5.4)	
	12 (7.6)	6 (7.2)	6 (8.1)	0.161
	15 (9.6)	12 (14.5)	3 (4.1)	
Total number of illnesses, median (range)	2.0 (0–10)	3.0 (0–10)	2.0 (0–8)	**0.003**
Daily drug intake	0.0 (0.0–4.0)	0.0 (0.0–4.0)	0.0 (0.0–3.0)	0.081
Sleep quality (OVIEDO scale), median (range)	3.0 (0.0–4.0)	2.8 (0.4–4.0)	3.2 (0.0–4.0)	0.122
Alcohol use frequency, n (%) - Never - 2 to 4 times per year - Once a month or less - 2 to 3 times per week - 4 or more times per week				
	23 (14.6)	12 (14.5)	11 (14.9)	
	41 (26.1)	27 (32.5)	14 (18.9)	
	63 (40.2)	36 (43.4)	27 (36.4)	0.009
	27 (17.2)	6 (7.2)	21 (28.4)	
	3 (1.9)	2 (2.4)	1 (1.4)	
Smoking frequency, n (%) - Never - 2 to 4 a year - At least one monthly - 2 to 3 weekly - >4 weekly				
	130 (82.8)	71 (85.6)	59 (79.6)	
	8 (5.1)	5 (6.0)	3 (4.1)	
	5 (3.2)	2 (2.4)	3 (4.1)	0.369
	5 (3.2)	3 (3.6)	2 (2.7)	
	9 (5.7)	2 (2.4)	7 (9.5)	
Consumption of the seven evaluated illicit substances, mean ± SD				
	0.0 (0.0–07)	0.0 (0.0–0.3)	0.0 (0.0–0.7)	0.091
**Psychological**				
Positive emotions, mean ± SD	2.8 ± 0.7	2.6 ± 0.8	2.9 ± 0.6	**0.015**
TEIQue: Assertiveness, mean ± SD	4.6 ± 1.3	4.5 ± 1.2	4.8 ± 1.3	0.183
TEIQue: Emotion identification, median (range)	5.4 (1.2–7.0)	5.4 (1.2–7.0)	5.4 (1.2–7.0)	0.931
TEIQue: Self-motivation, median (range)	5.2 (1.6–7.0)	5.2 (1.6–7.0)	5.2 (2.2–7.0)	0.272
SOC_13: Comprehensibility, median (range)	4.4 (1.6–7.0)	4.2 (1.6–6.6)	4.8 (2.0–7.0)	**0.033**
SOC_13: Manageability, mean ± SD	4.3 ± 1.4	4.1 ± 1.4	4.5 ± 1.4	0.078
SOC_13: Meaningfulness, median (range)	5.3 (1.8–7.0)	5.3 (1.8–7.0)	5.3 (2.8–7.0)	0.895
GAD-7, median (range)	0.9 (0.0–3.0)	1.0 (0.0–3.0)	0.9 (0.0–3.0)	0.405
Mini-ECCA, median (range)	7.0 (2.0–12.0)	8.0 (3.0–12.0)	6.5 (2.0–12.0)	0.083
PHQ-9, median (range)	0.6 (0.0–2.7)	0.7 (0.0–2.7)	0.4 (0.0–2.1)	**0.029**
SSS-8, median (range)	1.6 (1.0–2.9)	1.8 (1.0–2.9)	1.5 (1.0–2.8)	**0.004**
PSS-10, mean ± SD	1.5 ± 0.7	1.6 ± 0.8	1.4 ± 0.6	**0.048**
Traumatic events in life, median (range)	0.4 (0.0–2.0)	0.4 (0.0–1.2)	0.4 (0.0–2.0)	0.605
PWBS: Self-acceptance, median (range)	4.8 (1.0–6.0)	4.8 (1.0–6.0)	5.0 (2.0–6.0)	0.075
PWBS: Autonomy, median (range)	4.3 (1.5–6.0)	4.2 (1.5–6.0)	4.5 (2.2–6.0)	**0.020**
PWBS: Environmental mastery, Mean ± SD	4.6 ± 1.0	4.47 ± 1.1	4.71 ± 0.9	0.136
PWBS: Positive relations, median (range)	4.8 (1.0–6.0)	5.0 (1.0–6.0)	4.7 (1.8–6.0)	0.475
PWBS: Life purpose, median (range)	5.0 (1.4–6.0)	4.8 (1.4–6.0)	5.0 (1.6–6.0)	0.101
PWBS: Personal growth, median (range)	5.7 (1.3–6.0)	5.7 (1.3–6.0)	5.7 (3.0–6.0)	0.323
**Biochemicals**				
Leukocytes (1 × 10^3^/uL), median (range)	5.8 (3.4–11.0)	5.9 (3.4–11.0)	5.8 (3.5–8.5)	0.524
Lymphocytes (1 × 10^3^/uL), median (range)	1.8 (0.8–3.5)	1.7 (0.9–3.0)	1.8 (0.8–3.5)	0.456
Monocytes (1 × 10^3^/uL), median (range)	0.2 (0.1–0.4)	0.2 (0.1–0-4.0)	0.2 (0.1–0.4)	0.105
Granulocytes (1 × 10^3^/uL), median (range)	3.8 (1.9–7.9)	3.9 (2.0–7.9)	3.8 (1.9–6.3)	0.187
Platelets (1 × 10^3^/uL), mean ± SD	223.7 ± 47.0	236.5 ± 48.9	209.4 ± 40.7	**<0.001**
Hemoglobin (g/dL), median (range)	13.4 (7.3–19.2)	12.7 (7.3–17.4)	14.5 (12.9–19.2)	**<0.001**
Triglycerides (mg/dL), median (range)	97.8 (21.7–625.8)	97.6 (23.7–357.9)	99.6 (21.7–625.8)	0.256
Cholesterol (mg/dL), mean ± SD	185.1 ± 38.1	180.8 ± 33.7	189.9 ± 42.1	0.140
Glucose (mg/dL), median (range)	88.5 (59.4–232.0)	83.8 (59.4–120.6)	92.3 (61.6–232.0)	**<0.001**
Urea (mg/dL), median (range)	27.0 (10.0–143.7)	26.2 (14.2–143.7)	27.0 (10.0–58.7)	0.306
Global DNA methylation (%), median (range)	0.44 (0.0–2.1)	0.43 (0.0–1.9)	0.50 (0.1–2.1)	**0.045**
**Levels of inflammatory and oxidative stress biomarkers**				
TNF-α (pg/mL), median (range)	177.2 (15.6–1000.0)	62.9 (15.6–1000.0)	403.5 (15.6–1000.0)	0.132
IL-8 (pg/mL), median (range)	9.5 (7.8–462.0)	9.8 (7.8–462.0)	9.4 (7.8–380.5)	0.259
IL-6 (pg/mL), median (range)	5.0 (3.1–329.0)	6.2 (3.1–329.0)	4.2 (3.1–23.3)	**0.006**
IL-1β (pg/mL), median (range)	13.4(7.8–1000.0)	13.7 (7.8–1000.0)	13.3 (8.3–27.4)	0.410
IL-10 (pg/mL), median (range)	61.1 (7.8–1741.6)	43.3 (7.8–1741.6)	105.8 (7.8–1741.6)	0.399
8-Isoprostane (pg/mL), median (range)	296.8 (230.1–418.3)	291.0 (230.1–418.3)	300.9 (246.4–364.4)	0.175
8-OHdG, median (range)	1.9 (0.6–10.0)	1.9 (0.6–10.0)	2.0 (1.1–2.9)	0.218
**Anthropometrics and blood pressure**				
BMI, median (range)	25.6 (16.4–39.9)	25.9 (16.4–39.9)	25.2 (18.7–38.9)	0.783
WHR, median (range)	0.8 (0.7–1.2)	0.8 (0.7–1.0)	0.9 (0.7–1.2)	**<0.001**
Systolic BP (mmHg), median (range)	112.0 (80.0–164.0)	106.0 (80.0–155.0)	120.0 (90.0–164.0)	**<0.001**
Diastolic BP (mmHg), median (range)	77.0 (57.0–120.0)	75.0 (57.0–106.0)	79.0 (62.0–120.0)	**0.025**

Quantitative variables are expressed as median and range and mean ± standard deviation (SD). Qualitative variables are expressed as frequency and percentage. Comparisons between sexes were performed with chi-squared and Fisher exact tests for qualitative variables and with T-test and Mann–Whitney U test for quantitative ones. Statistically significant values are indicated by *p* < 0.05. Monthly income (MXN: Mexican pesos): 5 categories, from low to very high; smoking and alcohol consumption frequency were measured, from 0 to 4 (never to more than 4 times in a week); sleep quality (OVIEDO scale), from 0 to 4 (low quality to high quality); positive emotions (PST scale), from 1 to 5 (never to almost always); subscales for emotional intelligence (TEIQue scale): assertiveness, emotion identification, self-motivation, from 1 to 7 (totally disagree to totally agree); subscales for sense of coherence scale (SOC-13): comprehensibility, manageability, and meaningfulness from 1 to 7 (never or almost never to frequently); generalized anxiety disorder (GAD-7 scale), from 0 to 3 (never to almost all days); quality of food intake (Mini-ECCA scale), from 0 to 12 (very low quality to very high quality); depression with Patient Health Questionnaire (PHQ-9) from 0 to 3 (very low to very high), somatic symptoms with the Somatic Symptoms Scale (SSS-8) from 1 to 3 (low to high); Perceived Stress Scale (PSS-10), from 0 to 3 (never to frequently); traumatic events in life: average of the frequency of 37 different traumatic events, from 0 to 3 (never to more than twice); subscales for Psychological Well-Being Scale (PWBS): self-acceptance, autonomy, positive relations, life purpose, personal growth, from 1 to 6 (totally disagree to totally agree); TNF-α: tumor necrosis factor alpha; IL: interleukin; 8-OHdG: 8-hydroxy-2-deoxyguanosine; BMI: body mass index; WHR: waist-to-hip ratio; and BP: blood pressure.

**Table 2:** Significant bivariate correlations between the studied variables and global DNA methylation levels.

Variables	Total Sample	Women	Men
	N = 157	N = 83	N = 74
**Sociodemographics**			
Age	−0.181 *	—	−0.333 **
Having a romantic partner	—	—	−0.295 *
Having children	−0.169 *	—	−0.242 *
Employed	—	—	−0.236 *
Monthly income	0.197 *	—	—
Free time	0.190 *	—	0.269 *
Daily drug intake	−0.173 *	−0.243 **	—
**Levels of inflammatory and oxidative stress biomarkers**			
8-Isoprostane	0.160 *	—	—
TNF-α	0.210 **	—	0.252 *
IL-8	0.265 **	—	0.368 **
IL-10	0.211 **	—	0.268 *
**Biochemicals**			
Hemoglobin	—	—	−0.269 *
Granulocytes	−0.192 *	—	−0.225 ^†^
Triglycerides	−0.157 ^†^	—	−0.240 *
**Anthropometrics and blood pressure**			
BMI	−0.292 **	−0.244 *	−0.362 **
WHR	—	—	−0.336 **
Systolic BP	—	—	−0.232 *
Diastolic BP	−0.163 *	—	−0.295 *

* *p* < 0.05, ** *p* < 0.01. Correlations were performed with Spearman correlation tests. † This variable shows a marginal level of statistical significance, between 0.05 and 0.1. TNF-α: Tumor Necrosis Factor alpha; IL: interleukin; BP: blood pressure; BMI: body mass index; and WHR: waist-to-hip ratio.

**Table 3:** Multivariate regression analysis for global DNA methylation in overall sample and stratified by sex.

Variables	B	Beta Coefficient	Significance	Change in R^2^	Tolerance
**Total sample**					
Constant	0.888	—	0.000	—	—
IL-8	0.001	0.283	0.000	0.123	0.898
Free time	0.025	0.247	0.001	0.065	0.930
BMI	−0.007	−0.139	0.078	0.041	0.826
Male sex	0.110	0.210	0.005	0.025	0.913
Diastolic BP	−0.005	−0.193	0.015	0.028	0.822
TEIQue: Assertiveness	−0.035	−0.169	0.025	0.026	0.914
**Women**					
Constant	0.457	—	0.054	—	—
BMI	−0.010	−0.247	0.019	0.074	0.908
Daily drug intake	−0.046	−0.214	0.041	0.048	0.913
8-Isoprostane	0.002	0.288	0.006	0.045	0.939
PSS-10	−0.159	−0.591	<0.001	0.031	0.425
SOC-13: comprehensibility	−0.058	−0.398	0.006	0.052	0.486
Smoking frequency	−0.057	−0.243	0.024	0.061	0.871
SSS-8	0.114	0.229	0.069	0.033	0.632
**Men**					
Constant	1.199	—	<0.001	—	—
SOC-13: comprehensibility	−0.056	−0.245	0.007	0.372	0.845
IL-8	0.002	0.524	<0.001	0.082	0.945
Diastolic BP	−0.009	−0.297	0.001	0.051	0.986
Monthly income (MXN)	0.071	0.241	0.007	0.045	0.879
Having a romantic partner	−0.094	−0.152	0.074	0.032	0.934
Free time	0.016	0.152	0.089	0.019	0.846

R of the models, total: 0.555, women: 0.587, and men: 0.776.

## Data Availability

The databases generated in this study and statistical analyses are available by request to the author: aniel.brambila@academicos.udg.mx.

## Supplementary Materials

The following supporting information can be downloaded at: https://www.mdpi.com/article/10.3390/biom15091271/s1, File S1:Items included in the emotional intelligence subscales of the TEIQUE scale.
