# Supplementary material for: Association of Inflammatory and Oxidative Stress Biomarkers Adjusted by Personal, Psychological, Biochemical, Anthropometric, and Physiological Variables with Global DNA Methylation in a Sample of Mexican Individuals
Source: Biomolecules. 2025 Sep 2;15(9):1271. doi: 10.3390/biom15091271 (PMC12466971; doi:10.3390/biom15091271)
Supplement: Supplementary file 1 [file biomolecules-15-01271-s001.zip › biomolecules-3794226-supplementary.pdf]

Items included in the emotional intelligence subscales of the TEIQUE scale

Instruction: Please indicate the degree of agreement or disagreement with the following statements. Answer options: 1: absolutely disagree- 7: absolutely agree

Self-motivation

1.- On the whole, I'm a highly motivated person

2.- Sometimes, it feels like I'm producing a lot of good work effortlessly

3.- I normally find it difficult to keep myself motivated

4.- I tend to get a lot of pleasure just from doing something well

5.- I lose interest in what I do quite easily

Emotion identification

1.- I often find it difficult to recognize what emotion I am feeling

2.- I´m never really sure what I´m feeling

3.- Many times, I can´t figure out what emotion I´m feeling

4.- On the whole, I find it difficult to describe my feelings

5.- Most of the time, I know exactly why I feel the way I do

Assertiveness

1.- When I disagree with someone, I usually find it easy to say so

2.- I tend to "back down" even if I know I´m right

3.- I usually find it difficult to express myself clearly

4.- I would normally defend my opinions even if it meant arguing with important people

5.- I tend to speak well and clearly

6.- I often find it difficult to stand up for my right
